# Afatinib and its encapsulated polymeric micelles inhibits HER2-overexpressed colorectal tumor cell growth *in vitro* and *in vivo*

**DOI:** 10.18632/oncotarget.2050

**Published:** 2014-06-01

**Authors:** Siao-Syun Guan, Jungshan Chang, Chun-Chia Cheng, Tsai-Yueh Luo, Ai-Sheng Ho, Chia-Chi Wang, Cheng-Tien Wu, Shing-Hwa Liu

**Affiliations:** ^1^ Institute of Toxicology, College of Medicine, National Taiwan University, Taipei, Taiwan; ^2^ Institute of Nuclear Energy Research, Atomic Energy Council, Taoyuan, Taiwan; ^3^ Graduate Institute of Medical Sciences, College of Medicine, Taipei Medical University, Taipei, Taiwan; ^4^ Division of Gastroenterology, Cheng Hsin General Hospital, Taipei, Taiwan; ^5^ Division of Hepatology, Taipei Tzu Chi Hospital, Buddhist Tzu Chi Medical Foundation and School of Medicine, Tzu Chi University, Hualien, Taiwan; ^6^ Department of Medical Research, China Medical University Hospital, China Medical University, Taichung, Taiwan; ^7^ Department of Pediatrics, National Taiwan University Hospital, Taipei, Taiwan

**Keywords:** colorectal cancer, HER2, afatinib, micelles

## Abstract

Colorectal cancer (CRC) is known as a common malignant neoplasm worldwide. The role of EGFR/HER2 in CRC is unclear. Afatinib is an irreversible EGFR/HER2 inhibitor. There were few studies of afatinib on CRC. Here, we investigated the protein levels/expressions of HER2 in sera and tumors from CRC patients and the therapeutic effect of afatinib on HER2-overexpressed CRC *in vitro* and *in vivo*. The increased HER2 levels were detected in the collected sera and tumors of patients with CRC. The serological HER2 levels were correlated with the tumor HER2 expressions in patients. Afatinib also inhibited the HER2-positive tumor cell growth and caused apoptosis in HER2-overexpressed human colorectal cancer HCT-15 cells but not in low HER2 expressed human gastric cancer MKN45 cells. *In vivo* study showed that afatinib reduced tumor growth in HER2-overexpressed xenografts. Moreover, afatinib-encapsulated micelles displayed higher cytotoxic activity in HCT-15 cells and were more effective for tumor growth suppression in HCT-15-induced tumor xenografts than afatinib performance alone. Taken together, these findings suggest that higher serum HER2 levels reflect the higher HER2 contents in tumors of CRC patients, and the improved afatinib-encapsulated micelles possess high therapeutic efficacy in HER2-overexpressed CRC *in vitro* and *in vivo*.

## INTRODUCTION

Colorectal cancer (CRC) is a major worldwide health problem with high incidence and mortality [1-3]. In present, CRC accompanied with higher mortality is due to the disease that is frequently diagnosed in the advanced stage without reliable biomarkers. In order to decrease the mortality of CRC, searching for CRC biomarkers is urgent and important. In advance, developing targeted therapy can also effectively reduce mortality. In the past, chemotherapy has been shown to be an efficient strategy for tumor therapy, but is still not capable of preventing recurrence of CRC in patients [4]. Monoclonal antibodies and other biologicals, targeting tumor-associated proteins and blocking essential processes of the tumor, were extensively studied. A crucial step in this process is the identification of tumor specific proteins that can be targeted by these compounds.

Human epidermal growth factor receptor 2 (HER2/ErbB2) is a receptor tyrosine kinase member of the epidermal growth factor receptor (EGFR) family [5, 6]. HER2/ErbB2 contains an extracellular ligand binding domain, a transmembrane region, and an intracellular tyrosine kinase domain [7]. Kinase activity of HER2 is activated upon receptor dimerization, either with the homo- or hetero-dimerization [8]. Till now, the specific biological ligands to HER2 have not been identified yet. The conformational change and auto-phosphorylation of the intracellular domain of HER2 can lead to downstream signaling via mitogen-activated protein kinase, phosphoinositide 3-kinase, phospholipase C, protein kinase C, and signal transducer and activator of transcription for mediating tumor growth, differentiation, and survival [9-13]. HER2 overexpression leads to increased proliferation, angiogenesis, and reduced apoptosis. The overexpressed HER2 has been discovered in a number of human cancers, including in breast cancer [14-16], gastric cancer [17], ovarian cancer [18], and salivary gland carcinoma [19]. HER2 has attracted considerable attention in breast cancer and gastric cancer, where it has been targeted successfully in the treatment of patients with advanced stage [20, 21]. In both cancers, trastuzumab, a monoclonal antibody targeting HER2, has been shown to significantly improve therapeutic efficacy and survival in patients with HER2-postive tumors [20, 21]. However, HER2 as a therapeutic target in CRC has been investigated with controversial results [22-25]. The role of EGFR/HER2 in CRC still remains to be clarified.

Afatinib (BIBW 2992), an irreversible EGFR/HER2 inhibitor, has been shown to be effective in the elimination of cancer cells with *HER2* gene mutations in preclinical lung cancer models [26]. Several clinical trials of afatinib have been investigated in patients with advanced solid tumors [27]. In a clinical trial, the clinical activity of afatinib has been shown in patients with lung adenocarcinoma with *HER2* gene mutation [28]. However, the therapeutic potential of afatinib on HER2-overexpressed CRC remains to be clarified. In this study, we aimed to investigate the levels of HER2 in sera and tumors from CRC patients and the therapeutic effects of afatinib on CRC with high HER2 expression *in vitro* and *in vivo*. Moreover, nanoparticulate drug delivery systems have attracted increasing attention for diagnostic and therapeutic applications in cancers [29, 30]. Nano-carriers enable the preferential delivery of chemotherapeutic agents into the tumor foci due to the enhanced permeability and retention (EPR) [31]. Micelles are of particular interest in tumor applications due to their suitable size, biocompatibility, and hydrophobic core, which is emerging as powerful drug delivery vehicles for loading hydrophobic drugs [32]. Here, we also tried to test whether afatinib-encapsulated polymeric micelles (afatinib/micelles) can increase the therapeutic effect of afatinib in HER2-overexpressed tumors.

## RESULTS

### HER2 levels in serum and tumor tissue of CRC patients

**Figure 1:**
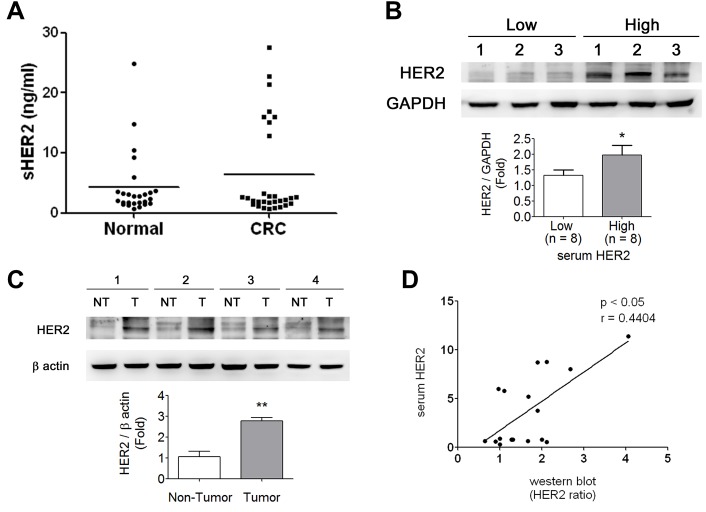
HER2 levels in sera and tumors of CRC patients (A) Serological HER2 (sHER2) highly expressed in ~25% (8/31) of CRC patients determined by an ELISA assay. (B) The HER2 expressions in CRC tumor tissues responding to the sHER2 levels. The tissues were chosen from three high- and low-expressional sHER2 of CRC patients for Western blotting. Protein levels were quantified by densitometry and normalized by GAPDH levels. Data are presented as means ± SEM (n = 3). *P< 0.05, high versus low sHER2. (C) The expressions of HER2 in CRC tumors and non-tumors from the individual patients with high sHER2 levels. Protein levels were quantified by densitometry and normalized by GAPDH levels. The data are presented as means ± SEM (n = 4). *P< 0.05, tumor versus non-tumor. (D) The sHER2 levels were correlated with tissue HER2 expressions in CRC patients. *p < 0.05, tumor HER2 versus sHER2.

There were about 25% (8/31) of CRC patients showing high-level sHER2 more than the average of HER2 concentration in disease group (Figure 1A), which was consistent with the results found in breast cancer patients [14-16]. The clinical characteristics of CRC patients were shown in Table 1. Moreover, protein expressions of HER2 in tumor tissues from patients with low- and high-level sHER2 were detected. As shown in Figure 1B, the protein expressions of HER2 in tumors were higher in patients with high-level sHER2 than in patients with low-level sHER2. The protein expressions of HER2 were also measured in tumor and non-tumor tissues from patients with high-level sHER2. The results showed that higher HER2 expressions were observed in tumor tissues than in non-tumor tissues (Figure 1C). We also observed that the levels of sHER2 are positively correlated with that of tumor HER2 in CRC patients (*p*<0.05, Figure 1D), considering that measurement of sHER2 may be used to reflect the levels of HER2 in tumors as a reference during cancer therapy.

**Table 1:** The clinical characteristics of normal volunteers and patients with colorectal cancer

	Normal volunteers (n = 26)	CRC Patients (n = 31)
Age (years)	51.5 ± 11.9	60.7 ± 12.8
Gender		
Male (number)	16	16
Female (number)	10	15
Body weight (kg)	66.3 ± 12.2	64.1 ± 10.5
BMI (kg/m^2^)	24.3 ± 3.7	24.8 ± 2.8
Serum CEA (ng/dL)		
<1	12	4
1-5	13	15
>5	1	12
Smoker (number)	1	7
Non-smoker (number)	25	24
Moderate or heavy drinker (number)	3	1
Light or non-drinker (number)	23	30

Abbreviations: BMI, body mass index; CEA, carcinoembryonic antigen.

### Cytotoxic effects of afatinib on tumor cells with or without HER2 overexpression

**Figure 2:**
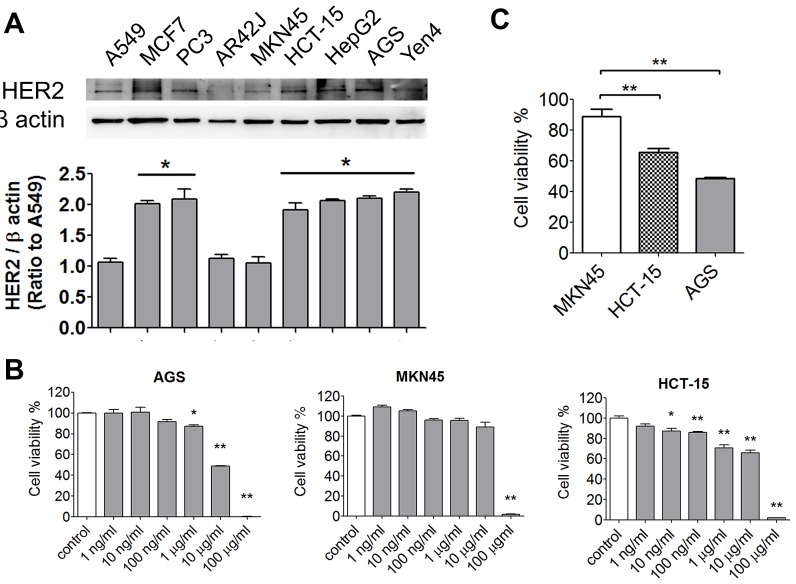
Effects of afatinib on cell viability of cancer cells with or without HER2-overexpression (A) The expressions of HER2 in various tumor cells. The lung (A549), breast (MCF-7), prostate (PC-3), gastric (AGS), colorectal (HCT-15), and hepatocellular (HepG2) tumor cells were chosen to detect HER2 levels determined by Western blotting. Protein levels were quantified by densitometry and normalized by β-actin levels. The data are presented as means ± SEM (n ≥ 4). *P< 0.05, versus A549 cells. (B) Cell viability assay. AGS, MKN45, and HCT-15 cells were treated with afatinib (1 ng-100 μg/ml) for 24 h. Data were presented as means ± SEM (n = 5). *P< 0.05, **P< 0.01, versus control. (C) Comparing with the cell viability in AGS, MKN45 and HCT-15 under afatinib (10 μg/ml) treatment. The data are presented as means ± SEM (n ≥ 5). **P< 0.01, versus MKN45 cells.

**Figure 3:**
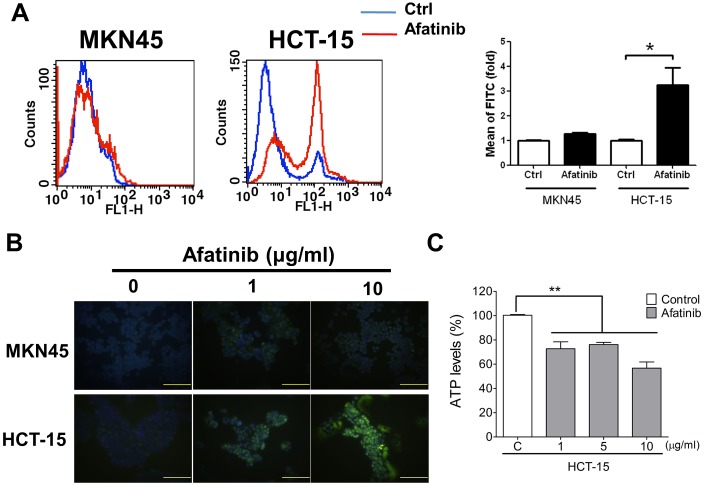
Afatinib causes cell apoptosis and mitochondrial toxicity in HER2-overexpressed CRC cells Cell apoptosis were determined by flow cytometric assay (AnnexinV and Propidium Iodide staining) (A) or TUNEL assay (B) in MKN45 and HCT-15 cells treated with afatinib (A, 10 μg/ml; B, 1 and 10 μg/ml) for 24 h. In A, data are presented as means ± SEM (n = 5). *P< 0.05, versus control. In B, the total nuclei were stained with 4’, 6-diamino-2-phenylindole (DAPI). Magnification: 400×, scale bar: 50 μm. (C) Mitochondrial dysfunction assay in afatinib-treated MKN45 and HCT-15 cells. Cells were treated with afatinib (1-10 μg/ml) for 24 h. Data are presented as means ± SEM (n = 5). **P< 0.01, versus control.

In order to establish an HER2-overexpressed cell model, the protein expressions of HER2 were detected in several kinds of cancer cells, including breast (MCF-7), prostate (PC-3), gastric (AGS and MKN45), colorectal (HCT-15), lung (A549), hepatocellular (HepG2), and esophageal (Yen4) tumor cells. As shown in Figure 2A, overexpressed HER2 was found in MCF-7, PC-3, HCT-15, HepG2, AGS, and Yen4 cells. To investigate the specific therapeutic effects of afatinib in tumors, AGS and HCT-15 cells were chosen as high-expression HER2 models, whereas MKN45 as a low-expression HER2 model. Compared to MKN45 cells, the tumor cell viabilities were significantly decreased by lower concentrations of afatinib (10 ng/ml-10 μg/ml) in HER2-overexpressed HCT-15 and AGS cells (Figure 2B). The cell viabilities of AGS and HCT-15 were significantly decreased by 10 μg/ml of afatinib about 20% and 40%, respectively (Figure 2C). Moreover, afatinib significantly increased cell apoptosis in HCT-15 cells as compared with MKN45 cells determined by Annexin V-FITC/PI double-labeled flow cytometry (Figure 3A) and TUNEL assay (Figure 3B). Then, we further observed whether mitochondrial dysfunction resulted in afatinib-induced apoptosis. Because the majority of ATP production takes place in the mitochondrial the ATP production is an indicator for detecting mitochondrial function. As shown in Figure 3C, intracellular ATP levels were significantly decreased in afatinib-treated HCT-15 cells.

### Afatinib inhibits tumor growth in a xenograft mouse model

**Figure 4:**
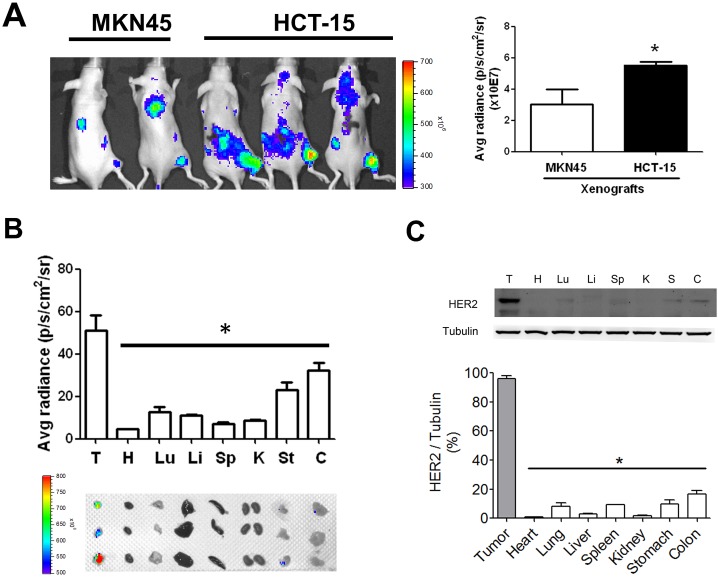
HER2 imaging in the tumors and organs of HCT-15-induced xenografts in mice (A) The HER2 imaging detection in HCT-15 xenograft model. (A) HCT-15 cells (2 × 10^6^) were subcutaneously inoculated into the right flank of nude mice for establishing a HER2-overexpressed xenograft model, whereas MKN45-induecd xenografts were as a low HER2-expressed model. The HER2 antibodies labeled with fluorescent were injected into mice for 24 h and detected by using *In vivo* imaging system (IVIS). Data are presented as means ± SEM (n ≥ 3). *P< 0.05, versus MKN45. (B) The HER2-distributed imaging in various organs of HCT-15 xenograft mice. After whole animals imaging assay, HCT-15 xenograft mice were sacrificed and acquired tumor (T), heart (H), lung (Lu), liver (Li), spleen (Sp), kidney (K), stomach (St), and colon (C) for fluorescent imaging assay by using *In vivo* imaging system (IVIS). The Top panel showed quantitation of fluorescent signals. Button panel showed the imaging of HER2 in various organs. (C) The expression of HER2 in various organs of HCT-15 xenograft mice. Following organs fluorescent imaging assay, all organs were homogenized for western blotting to compare the expressions of HER2. The overexpressed characteristic in tumors was consistent with the results demonstrated in HER2 fluorescent images. All data are presented as means ± SEM (n = 3). *P< 0.05, versus tumor.

**Figure 5:**
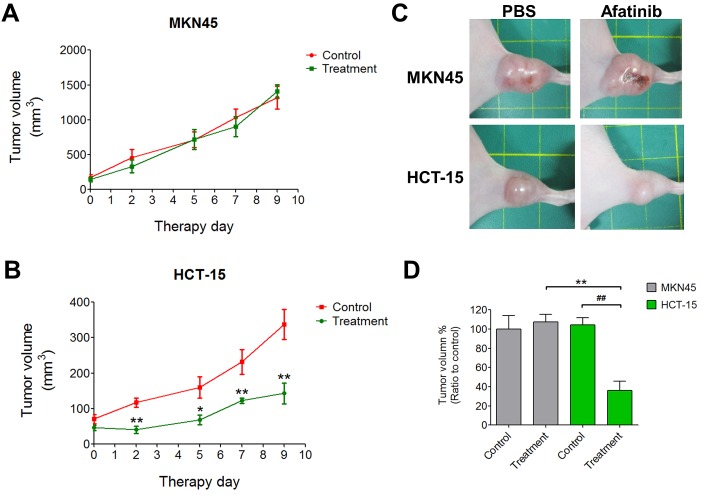
Afatinib inhibits HER2-postive tumors in a xenograft mouse model (A-C) MKN45 or HCT-15 cells (2 × 10^6^) were subcutaneously inoculated into the right flank of nude mice (n = 5 per treatment group). Tumors were established for 14 days before the afatinib (5 mg/kg) treatment. Tumor volume was calculated by the formula: tumor volume [mm^3^] = (length [mm]) × (width [mm])^2^. Data are presented as means ± SEM (n = 5). **p < 0.01, versus control. (D) The ratios of tumor reduction in MKN45- and HCT-15-induced xenografts in mouse with or without afatinib treatment. Data are presented as means ± SEM (n = 5). **p < 0.01, versus MKN45. ^##^*p*< 0.01, versus control.

Next, the images of HER2 expression were detected in MKN45- and HCT-15-induced xenografts in mice. The fluorescence signals were significantly increased in the HCT-15-induced xenograft mice as compared with MKN45-induced xenografts (Figure 4A). The distributions of HER2 in several organs of HCT-15-induced xenografts were also detected. Tumor tissue possessed higher fluorescence signal as compared with other organs (Figure 4B). The protein overexpression of HER2 was also observed in tumor tissue determined by Western blotting, which was consistent with the results of fluorescence imaging (Figure 4C). Furthermore, to evaluate the antitumor effect of afatinib *in vivo*, the xenograft mouse model transplanted with HCT-15 cells (high-expression HER2) or MKN45 cells (low-expression HER2) was established. Afatinib had no inhibitory effect on the tumor growth of MKN45-induced xenografts (Figures 5A and 5C). However, the tumor growth of HCT-15-induced xenografts was significantly inhibited by afatinib treatment (Figures 5B and 5C). The tumor volume was also significantly decreased by 60% in afatinib-treated HCT-15-induced xenografts; whereas no effect of afatinib was observed in MKN45-induced xenografts (Figure 5D).

### Afatinib encapsulated by polymeric micelles increases the therapeutic efficacy of afatinib

**Figure 6:**
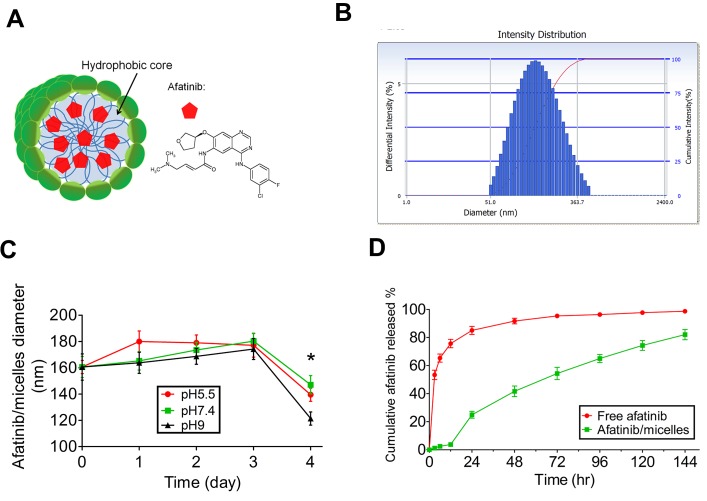
The characteristics of afatinib/micelles (A) Afatinib/micelles were illustrated, where afatinib was encapsulated in the hydrophobic center of polymeric micelles. (B) The particle sizes of afatinib/micelle were determined using DLS. The average of afatinib/micelles was measured as 160 ± 32 nm. (C) *In vitro* stability of afatinib/micelles. The size distribution of afatinib/micelles was observed at different buffer conditions (pH = 5.5, 7.4, 9) for 4 days at 37°C and measured by using DLS. Data are presented as means ± SEM (n = 5). *p < 0.05, versus Day 0. (D) *In vitro* afatinib release behavior of afatinib/micelles and free afatinib. The equal concentration of afatinib/micelle or free afatinib solution (1 mg/ml) was injected into the dialysis bag and dialyzed against absolute alcohol at 37°C. The outer solution containing released afatinib was collected at predetermined time points. The amount of the released afatinib was measured using UV-Vis spectrophotometer at a 289 nm wavelength. Data are presented as means ± SEM (n = 5).

In order to improve the therapeutic efficacy of afatinib, the polymeric micelles-encapsulated drug system consisting of amphiphilic block copolymers and afatinib (afatinib/micelles) was synthesized (Figure 6A). The sizes of afatinib/micelles were 160 ± 32 nm and determined by a dynamic light scattering (DLS) (Figure 6B). To detect the stability of afatinib/micelles, the size of afatinib/micelles in various pH conditions (pH = 5.5, 7.4, and 9) was measured. The result indicated that the size of afatinib/micelles was no difference for 3 days and were decreased significantly after 4 days (Figure 6C). To observe the *in vitro* release kinetics of afatinib/micelles, the modified dialysis method was performed. The results showed that free afatinib exhibited a very fast release rate, whereas afatinib/micelles showed sustained release behavior (Figure 6D). In the first 24 hours, 85.2 ± 5.3% and 24.9 ± 4.6% of afatinib were released from the free afatinib and afatinib/micelles groups, respectively (P < 0.05). Furthermore, the measurement of afatinib/micelles release behavior showed that a time for 50% of afatinib release (t_1/2_) was approximately at 72 h and the majority of drug (>80%) was released in a 144-hour period.

**Figure 7:**
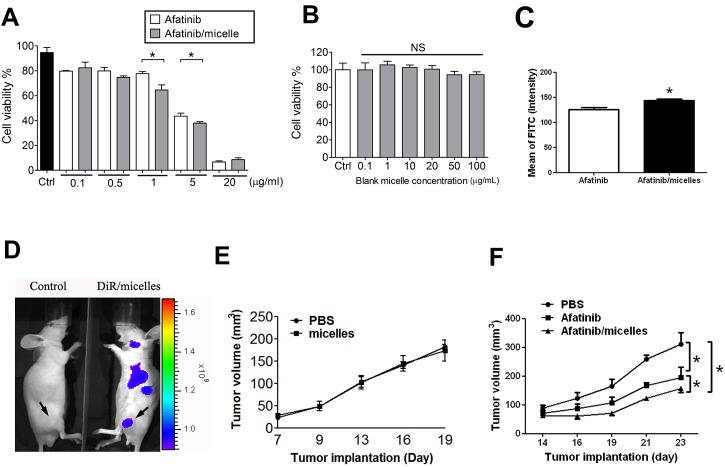
Afatinib/micelles increase the therapeutic efficacy of afatinib on CRC models (A-B) Cell viability was measured using WST-8 assay. HCT-15 cells were treated with afatinib or afatinib/micelles (0.1-20 μg/ml) (A) and blank micelle (0.1-100 μg/ml) (B) for 24 hrs. Data were presented as means ± SEM (n ≥ 5). *P< 0.05, versus afatinib. NS, Non-significant. (C) HCT-15 was treated with 10 μg/ml of afatinib or afatinib/micelles for 24 h, and then cells were stained AnnexinV and Propidium Iodide staining for apoptosis assay. (D) The distribution imaging of micelles in HCT-15-induced xenografts. In order to ensure the accumulative effect of micelles in tumors, the DiR/micelles were produced and injected into HCT-15-induced xenograft mice for 24 hrs. Then, the *In vivo* imaging system (IVIS) was performed to capture the imaging for detecting micelles distribution. (E) The effects of micelles alone on HCT-15-induced xenografts. Tumor volume was calculated by the formula: tumor volume [mm^3^] = (length [mm]) × (width [mm])^2^. All data are presented as means ± SEM (*n* = 5). (F) The therapeutic efficacy of PBS, afatinib alone, and afatinib/micelles in the HCT-15 xenograft model. Tumor volume was calculated by the formula: tumor volume [mm^3^] = (length [mm]) × (width [mm])^2^. All data are presented as means ± SEM (*n* = 5). *P < 0.05, versus afatinib.

Afatinib/micelles treatment showed higher decrease in cell viability of HCT-15 cells than afatinib alone treatment (Figure 7A). However, the micelles alone treatment showed non-cytotoxicity in HCT-15 as compared to PBS treatment (Figure 7B). Furthermore, a flow cytometric analysis was performed to observe the level of apoptosis after afatinib/micelles or afatinib alone treatment in HCT-15 cells. The results indicated that cell apoptosis was significantly increased in afatinib/micelles-treated tumor cells as compared with afatinib alone treatment (Figure 7C). To understand the distribution of afatinib/micelles in *in vivo* xenografts, a fluorescent dye DiR, was encapsulated in micelles (DiR/micelles). The fluorescence images showed that micelles are accumulated in tumor of HCT-15-induced xenograft as compared with control mice (Figure 7D). The micelles alone treatment indicated that the growth of tumor tissues was no difference in HCT-15-induced xenografts as compared with PBS treatment (Figure 7E). Afatinib/micelles treatment could also significantly increase the inhibition of tumor growth in HCT-15-induced xenografts as compared with afatinib alone treatment (Figure 7F). These results indicated that micelles can carry afatinib into tumor tissues and consequently improve the therapeutic efficacy.

## DISCUSSION

In this study, we first evaluated HER2 as a CRC biomarker and found that serological HER2 (sHER2) levels are correlated with tumor tissue HER2 levels in clinical CRC patients. We also found that afatinib effectively inhibits tumor cell growth of CRC with high-expression HER2 *in vitro* and *in vivo*. Afatinib could cause cell apoptosis in HER2-overexpressed CRC cells through the induction of mitochondrial toxicity. In addition, afatinib-encapsulated polymeric micelles significantly increased the therapeutic efficacy of afatinib on HER2-overexpressed CRC *in vitro* and *in vivo*. These findings suggest that afatinib effectively inhibits HER2-overexpressed CRC cell growth and the afatinib/micelles significantly increase the accumulation of afatinib in tumor tissues and improve the therapeutic efficacy of afatinib in HER2-overexpressed CRC tumors.

HER2 is known as a critical target in therapy and diagnosis for breast cancer, which occurs in 20-30% of the breast cancer population. The levels of serum HER2 as well as tumor tissue HER2 have been detected to be increased in the patients with breast cancer [33]. In clinical practice,immunohistochemistry (IHC) and fluorescence in situ hybridisation (FISH) are the suggested methods to determine HER2 status in cancer specimens [33, 34]. Currently, FDA has approved several kits or assays of IHC or FISH to determine HER2 expression. Even though the techniques present highly standardized and semi-quantitative assay, IHC has been shown to have numerous limitations, including operative and illustrative difficulties without quantitative measurements, which may lead to the low reproducibility and accuracy. Moreover, the procedure of IHC is complicated and time-consuming, which are undesirable for detection in a lot of clinical samples. FISH is considered to be a “gold standard” technique with high sensitivity and accuracy in recognize HER2 expression in tumor samples, but it is expensive and lacks standardized quantitative system for distinguishing HER2 expression. In this study, we observed that the average serological HER2 (sHER2) levels are increased in CRC patients (~25% above average), which are correlated with tumor tissue HER2 expressions. These results implied that the serum HER2 levels determined by ELISA assay may be a profitable and invasive tool for diagnosing HER2 expression in CRC patients.

Afatinib is known as a first-line FDA-approved drug for the treatment of metastatic non-small cell lung carcinoma (NSCLC) with EGFR mutations, which is a tyrosine kinase inhibitor (TKI) not only against EGFR mutations but also inhibit HER2-overexpressed breast cancer. Lin *et al.* observed that there are about 46% patients with overexpressed HER2 achieving benefit from afatinib treatment in the phase II and III trials [35]. Moreover, a previous study has shown that afatinib causes antitumor activity in HER2-overexpressed gastric cancer *in vivo* [36]. Li *et al*. have indicated that afatinib shows lower half-maximal inhibitory concentrations for *in vitro* potency against HER2 than that of lapatinib, another dual tyrosine kinase inhibitor [26]. In this study, we observed that afatinib causes cytotoxicity in HER2-overexpressed HCT-15 cell, and suppressed HCT-15-induced tumor growth *in vitro* and *in vivo*. Therefore, afatinib may be a potential agent in Her2-postive colorectal cancer therapy.

In this study, we showed that afatinib can induce cell apoptosis in HER2-overexpressed human colorectal cancer cells, which is consistent with the results in HER2-postive gastric and breast cancer cells [36, 37]. Lapatinib has been shown to inhibit the HER2 downstream signaling pathways RAF/MEK/ERK (ERK cascade) and PI3K/AKT/mTOR and lead to up-regulations of BIM (BCL-2–interacting mediator of cell death) and BID (BCL-2 antagonist of cell death) in HER2-overexpressed breast cancer cells [38]. In addition, lapatinib also induced reactive oxygen species-dependent endoplasmic reticulum stress to decrease mitochondrial function in breast cancer cells [38]. Our results also showed that afatinib causes the mitochondrial toxicity in HER2-overexpressed human colorectal cancer cells (HCT-15). As we know, the mitochondrial toxicity could induce permeabilization of the mitochondrial outer membrane and then triggers cytochrome c release for apoptosis induction.

Another goal in this study was to create the micelles-carried afatinib to improve the therapeutic efficacy of this drug. We found that afatinib encapsulated with polymeric micelles (afatinib/micelles) can improve the therapeutic efficacy in HER2-overexpressed HCT-15-induced tumors. Micelles have emerged as an important potential drug delivery vehicle for chemotherapy drugs and small molecule compounds in tumor therapy application. The therapeutic efficacy of these agents can be significantly improved when their circulation longevity and tumor site accumulation properties are increased through micelles encapsulation. The biocompatible micelles were suitable to carry hydrophobic drug such as afatinib for tumor therapy. Blanco *et al.* have demonstrated that β-lapachone encapsulated with polymeric micelles (β-lapachone/micelles) significantly suppress tumor volume and increase survival in a lung cancer model [39]. Currently, several polymeric micelles-formulated antitumor drugs were investigated in clinical trials, including paclitaxel, cisplatin, doxorubicin, and SN-38 [40-43]. Previous study has indicated that tumor vessels have large endothelial fenestrations ranging in size from 100 to 600 nm [44]. However, the large particle size (>220) will not be able to pass through the fenestrations between the tumor endothelial cells [45] and small particles (<10 nm) are rapidly excreted by the kidney [46]. The size of afatinib/micelles is about 92 nm, which accumulated in tumor tissues. Interestingly, we observed that afatinib/micelles (1-5 μg/ml) reveal more cytotoxicity than afatinib alone *in vitro*. The micelles may increase the uptake of afatinib through endocytosis in tumors, whereas afatinib may enter into tumor cells through diffusion [47]. Moreover, nanoparticles can reduce the drug resistance, hence to increase the therapeutic effect [48]. Therefore, in this study, afatinib/micelles produced not only the increased afatinib solubility in biology, but also the enhanced accumulation in tumors. Therefore, afatinib/micelles may be a potential drug system for HER2-overexpressed CRC therapy in clinical.

In this study, the micelles distribution imaging showed that afatinib/micelles accumulated not only in tumors but also in liver and spleen. Moreover, it has been indicated that nano-size particles such as liposome, dendrimer, and gold nanoparticle have been proposed to occur by uptake of the carrier by the mononuclear phagocyte system (MPS)/reticuloendothelial system (RES) recognition [49]. Even though the MPS uptake of nanoparticle caused rapid removal from the blood and accumulated in liver irreversibly, afatinib/micelles showed a less cytotoxicity response in normal liver cells and the expression of HER2 in liver was lower than in tumors (data not shown). The histological examination revealed that normal morphology in liver treated with afatinib/micelles was observed. Therefore, we considered that the afatinib micelles are less cytotoxicity in liver tissue.

The strength of this manuscript might be enhanced considerably if the studies focused on the clinical responses of the colorectal cancer patients to afatinib with some mechanistic details. However, at present, Food and Drug Administration in Taiwan has not yet permitted afatinib treatment in CRC patients. Therefore, it’s difficult to acquire the afatinib-treated samples from CRC patients for studying the mechanism on clinical responses of CRC patients to afatinib. Our *in vitro* study indicated that HER2 expression was significantly decreased in afatinib-treated HCT-15 cells (data not shown). Therefore, we suggested that afatinib might inhibit HER2 expression for decreasing cell proliferation in CRC cells and tumors. In this study, we attend to detect the efficiency of afatinib and afatinib/micelle in Her2-overexpressed CRC cells and tumors for a pre-clinical feasibility study.

In conclusion, we demonstrated that HER2 is a CRC therapeutic target, and the measurement of serum HER2 may be a potential tool for detecting HER2 expression in CRC. Afatinib can specifically inhibit HER2-overexpressed CRC cell growth *in vitro* and *in vivo*. Afatinib-encapsulated micelles displayed higher cytotoxic activity in HCT-15 cells and were more effective for tumor growth suppression in HCT-15-induced tumor xenografts than afatinib performance alone. Regarding afatinib/micelles as an anti-tumor drug in HER2-overexpressed CRC therapy, other HER2-overexpressed cancer such as gastric or ovarian tumor could be a candidate in the future.

## METHODS

### Acquisition of the tissues of colorectal cancer

Clinical samples were obtained from Cheng Hsin General Hospital, Taiwan and written informed consent and previous approval by the Institutional Review Board (CHGH-IRB-(240) 100-01) from CRC patients (n = 31) and healthy volunteer (n = 26). The pairs of tissues including tumors (T) and adjacent non-tumors (NT) from the individuals of CRC were captured followed by surgery. Tumor histopathology, grade, and stage were assigned according to the rules of American Joint Commission on Cancer Staging (AJCCS) system.

### Cell lines

Human lung adenocarcinoma epithelial cells (A549), human breast cancer cells (MCF7), human prostate cancer *cells (*PC3), rat pancreatic tumor cells (AR42J), human gastric cancer cells (MKN45), human colorectal carcinoma cells (HCT-15), human hepatocellular carcinoma cells (HepG2), human gastric adenocarcinoma *cells (*AGS) were purchased from the American Type Culture Collection (ATCC) and maintained in ATCC’s recommended culture medium with 10% of *fetal bovine serum*. The esophageal cancer cells (Yen4) were obtained from Cheng Hsin General Hospital, Taiwan and cultured in DMEM medium with 10% of *fetal bovine serum*. All cells were incubated at 37 °C and 5% CO_2_.

### Animals

Male nude mice were obtained from National Laboratory Animal Center, Taiwan. The 8-week-old mice were selected and housed on a 12-h light cycle at 22 °C and given food (mouse standard diet) or water *ad libitum*. All animal studies were approved by the institutive ethical review committee and were carried out in accordance with government regulations and NIH guidelines on the care and welfare of laboratory animals. All animals were sacrificed and then isolated the organs including brain, heart, liver, kidney, spleen, stomach and intestines after measuring body weight and tumor volume.

### Human Serum HER2 assay

Enzyme-linked immunosorbent assay (ELISA) system (eBioscience, USA) was performed to measure the human serum HER2 (sHER2) levels in the clinical serum samples (31 colorectal cancer patients and 26 control participants). Measurements were performed strictly according to the manufacturer’s instructions and quality control was ensured.

### Cellular viability assay

Cellular viability was determined by using WST-8 assay (Sigma-Aldrich, USA). The cells were cultured in 96-well plates at 37°C and 5% CO_2_. After 24 hours pre-incubation, each well (1×10^4^ cells) was treated with afatinib or afatinib-encapsulated polymeric micelles (afatinib/micelles) or micelles alone for 24 hours and then 10 μl of WST-8 solution was added to each well. After 3 hour-incubation at 37°C, the samples were measured using a microplate reader (Bio-Rad, USA).

### TUNEL assay

Cells were seeded in Millicell EZ slide (8 well) (Merck *Millipore, Germany)* with cultured medium for 12 hours at 37°C and 5% CO_2_. Then the cells were treated with afatinib or afatinib/micelles for 24 hours in 37°C and 5% CO_2_. After cell fixation, the cell apoptosis was measured by using the TUNEL assay system (Promega, USA) according to a manufacturer’s instruction.

### Mitochondrial function assay

Measurement of tumor cell mitochondrial function was performed in triplicate using the luminescence ATP detection system (PerkinElmer, USA). It is a cell-based assay method that employs a sequential addition to measure ATP as an indicator of mitochondrial function for predicting potential mitochondrial dysfunction as a result of xenobiotic exposure. Cells were cultured in 96-well plates at 37°C and 5% CO_2_. After 24 hours pre-incubation, each well (1×10^4^ cells) was treated with 10 μg/ml of afatinib for 24 hours. Cells were then lysed and used for ATP concentration measurements according to a manufacturer’s instruction. Results were normalized to protein content.

### Immunoblotting

Cells and tissues were lysed in the buffer containing 150 mM NaCl, 1% NP-40, 0.1% SDS, 50 mM Tris-HCl (pH8.0). The 15 g of protein samples was diluted in 2X Laemmli sample buffer (final concentrations: 75 mM Tris pH 6.8, 10% (v/v) glycerol, 2% SDS (w/v), 0.002% (w/v) bromophenol blue) and separated by the 10% sodium dodecyl sulfate polyacrylamide gel electrophoresis, and then transferred onto the Immobilon P membranes (Merck *Millipore, Germany*). These membranes were blocked in blocking buffer (Goal Bio, USA) in for 1 min at room temperature. Membranes were incubated with primary antibodies overnight at 4°C, and then washed four times in Tris buffered saline with 0.1% tween-20 (TBST) for 10 min. After washing, membranes were incubated with horseradish peroxidase-conjugated secondary antibody (diluted 1: 3000) for 1 hour at 4°C. The immunoreactive complexes were detected by ECL (Bio-Rad, USA) and monitored with a LAS-4000 mini (Fuji Film, Japan).

### Flow cytometry (FCM) analysis

MKN45 and HCT-15 cells were seeded at 8 × 10^5^ cells/ml in 6-cm dish, which were incubated either alone or in the presence of afatinib or afatinib/micelles at 10 μg/ml for 24 hours. After 24-hour incubation, media were removed and washed with PBS, and then incubated with 20 mM propidium iodide (PI) for 30 min. Apoptosis was evaluated by Annexin V-FITC staining with or without propidium iodide (PI). The cells were washed and harvested for flow cytometric analysis by FACSCalibur Flow Cytometer (BD Bioscience, USA).

### Afatinib/micelles synthesis and characterization

The 1,1’-dioctadecyltetramethyl indotricarbocyanine iodide (DiR) is a lipophilic carbocyanine dye. DiR-loaded micelles (DiR/micelles) were prepared via the co-solvent evaporation method as previously described [50]. Briefly, a mixture of 54 mg of MPEG-PCL and 6 mg of Mal-PEG-PCL in a ratio of 9:1 was dissolved in acetone with 3 mg of DiR dye (D/P = 1/20), and then added into saline while stirring with a rotor-stator device (VARIOMAG POLY 15, H+P Labortechnik AG, Germany). The organic solvent was evaporated while stirred overnight and then filtered through a Millex GS filter (pore size 0.22 μm) to remove non-incorporated drug crystals and copolymer aggregates. To produce afatinib/micelles, 10 mg of afatinib was dissolved in 1 mL of acetone and 20 μL of triethylamine (TEA) and stirred overnight. The afatinib solution was then mixed with 90 mg of MPEG-PCL and 10 mg of Mal-PEG-PCL (afatinib/PPLA ratio = 1/10). The mixed solution was added into saline, and the procedure described for making nanoparticles was performed. The mean diameters and polydispersity of micelles were measured by dynamic light scattering (DLS, DelsaTM Nano Particle Analyzer, Beckman Coulter, Fullerton, CA, USA). The free DiR and afatinib were removed through a Sephadex G-25 column and eluted with 0.15 M of NaCl buffer (pH = 7). The particle average size distribution of prepared afatinib/micelles was characterized by using a dynamic light scattering (DLS, DelsaTM Nano Particle Analyzer, Beckman Coulter, Fullerton, CA, USA).

### Afatinib/micelles stability and afatinib release *in vitro*

To investigate the stability of afatinib/micelles, the size distribution of afatinib/micelles was measured to observe the variation of particles diameter based on pH of the solvent. Briefly, the freeze-dried afatinib/micelles were dissolved in aqueous solvent with different pH: 5.5, 7.4, and 9 at 37°C. Then, we measured the changes in the diameter of afatinib/micelles in an aqueous solution over time (up to 4 days). Samples (300 μl) were collected from the stock solution and analyzed by using DLS. For afatinib release assay, one mg of freeze-dried afatinib/micelles were dissolved in PBS buffer (1 ml) and injected into a dialysis bag with the molecular weight cutoff value of 1 kDa, and dialyzed against 15 ml absolute alcohol and shaken at 37°C. The outer solution containing released afatinib was collected at predetermined time points and replaced with 15 mL of fresh absolute alcohol. The amount of the released afatinib was measured using UV-Vis spectrophotometer (V-650, JASCO, USA) at a 289 nm wavelength and calculated on the basis of a calibration curve using different concentrations of free afatinib in absolute alcohol. The experiment was carried out in triplicate.

### *In vivo* HER2 and micelles distribution imaging

The HER2 and micelles distribution imagings were performed in MKN45 or HCT-15 xenograft model. MKN45 or HCT-15 cells (2 × 10^6^) were subcutaneously (s.c.) inoculated into the right flank of nude mice (n = 5 per each group). Tumors were established for 10 days before the imaging assay. For *in vivo* HER2 imaging assay, the HER2-detected agent labeled with fluorescent (HER2Sense™ 645, PerkinElmer, USA) was *intravenously (i.v.) injected* into tail vein of MKN45 or HCT-15 xenograft model for 24 h. For *in vivo* micelles imaging assay, the DiR/micelles were *intravenously injected* into tail vein of HCT-15 xenograft mouse for 24 hours. *In vivo* imaging system (IVIS, PerkinElmer, USA) was performed to capture the imaging for detecting HER2 or micelles distribution. After whole body imaging assay, all animals were sacrificed and then isolated the organs including tumor, heart, lung, liver, spleen, kidney, stomach and intestines for IVIS detection. Then all organs were immediately frozen in liquid nitrogen. Tissues were stored at −80°C and analyzed for Western blotting within 3 days.

### *In vivo* tumor growth inhibition assay

*In vivo* antitumor effects of afatinib or afatinib/micelles were observed on male BALB/c Nude mice, which subcutaneously inoculated with MKN45 or HCT-15 cells (2 × 10^6^ /ml). Body weight and tumor appearance were observed once for every three days for 14 days. For the effect of afatinib on HER2 expression, mice were randomly distributed into two groups: vehicle control (n = 5) and afatinib (n = 5) in MKN45 or HCT-15 cell inoculated mice. Mice were treated once for every three days with either vehicle (normal saline) or afatinib (5 mg/kg body weight, i.v.) for 9 days. To detect the effects of micelles alone on CRC tumor growth, mice were randomly distributed in two groups: vehicle control (n = 5) and micelles alone (n = 5) in HCT-15 cell inoculated mice. Mice were treated once for every three days with either vehicle (normal saline) and micelles (5 mg/kg body weight, i.v.) for 9 days. Tumor growth was externally measured using a digital caliper, and tumor volumes were calculated using the formula (width^2^ × length), where the width represents the smaller tumor diameter. For comparing antitumor effects between afatinib and afatinib/micelles, mice were randomly distributed in three groups: vehicle control (n = 5), afatinib (n = 5), and afatinib/micelles (n = 5) in HCT-15 cell inoculated mice. Mice were treated once for every three days with either vehicle (normal saline), afatinib (5 mg/kg body weight, i.v.), and afatinib micelles (5 mg/kg body weight, i.v.) for 9 days. Tumor growth was externally measured using a digital caliper, and tumor volumes were calculated using the formula (width^2^ × length), where the width represents the smaller tumor diameter. Tumors were expatriated from mice at the end of the study and tumor weight was recorded.

### Statistical analysis

The Western blotting bands were quantified by densitometric analysis using Multi Gauge v3.2 software. Statistical analysis was performed using GraphPad Prism V5.01 software (GraphPad Software, Inc. USA). All analyses of rodent data with more than two groups were performed by ANOVA followed by post hoc analysis with Bonferroni’s test. Student’s *t* test was used to compare two groups where appropriate. Data were presented as mean ± SD. Differences between means were considered signification when *p* value <0.05.
